# Intelligent systems distribute memory across internal and embodied resources

**DOI:** 10.64898/2026.10.16.759765

**Published:** 2026-10-19

**Authors:** Ruiyi Zhang, Xaq Pitkow, Dora E Angelaki

## Abstract

Intelligent systems do not operate in isolation from the body, yet how bodily actions support internal computation remains unclear. Here we show that primates and artificial agents recruit eye movements to maintain beliefs about hidden goals when internal computation is constrained. In a naturalistic navigation task, both systems spontaneously tracked goals with their eyes after they disappeared from view. This strategy emerged in artificial agents only when internal memory was constrained, identifying resource constraints as a driver of memory externalization. Restricting gaze impaired performance in both systems, while natural behavioral variability and causal perturbations in agents established a direct influence of oculomotor state on internal beliefs about hidden goals. Across agents, varying the reliability of internal memory and gaze redistributed goal information between internal and embodied states. The same resource-allocation principle predicted the distribution of goal information in macaque parietal cortex, where greater reliance on gaze was accompanied by weaker gaze-independent neural representations. Together, these findings identify eye movements as embodied memory and reveal a convergent principle across intelligent systems: memory is allocated across internal and embodied resources according to their reliability.

## Full Text Availability

The license terms selected by the author(s) for this preprint version do not permit archiving in PMC. The full text is available from the preprint server.

